# Functional characterization of human pluripotent stem cell-derived cortical networks differentiated on laminin-521 substrate: comparison to rat cortical cultures

**DOI:** 10.1038/s41598-019-53647-8

**Published:** 2019-11-20

**Authors:** Tanja Hyvärinen, Anu Hyysalo, Fikret Emre Kapucu, Laura Aarnos, Andrey Vinogradov, Stephen J. Eglen, Laura Ylä-Outinen, Susanna Narkilahti

**Affiliations:** 1 Faculty of Medicine and Health Technology and BioMediTech, Tampere University, Tampere, Finland; 2 Institute of Biotechnology, HiLIFE, University of Helsinki, Helsinki, Finland; 3 Department of Biomedicine, Aarhus University, Aarhus, Denmark; 4 Danish Research Institute of Translational Neuroscience - DANDRITE, Aarhus University, Aarhus, Denmark; 5 Department of Applied Mathematics and Theoretical Physics, University of Cambridge, Cambridge, United Kingdom

**Keywords:** Extracellular recording, Induced pluripotent stem cells, Stem-cell differentiation

## Abstract

Human pluripotent stem cell (hPSC)-derived neurons provide exciting opportunities for *in vitro* modeling of neurological diseases and for advancing drug development and neurotoxicological studies. However, generating electrophysiologically mature neuronal networks from hPSCs has been challenging. Here, we report the differentiation of functionally active hPSC-derived cortical networks on defined laminin-521 substrate. We apply microelectrode array (MEA) measurements to assess network events and compare the activity development of hPSC-derived networks to that of widely used rat embryonic cortical cultures. In both of these networks, activity developed through a similar sequence of stages and time frames; however, the hPSC-derived networks showed unique patterns of bursting activity. The hPSC-derived networks developed synchronous activity, which involved glutamatergic and GABAergic inputs, recapitulating the classical cortical activity also observed in rodent counterparts. Principal component analysis (PCA) based on spike rates, network synchronization and burst features revealed the segregation of hPSC-derived and rat network recordings into different clusters, reflecting the species-specific and maturation state differences between the two networks. Overall, hPSC-derived neural cultures produced with a defined protocol generate cortical type network activity, which validates their applicability as a human-specific model for pharmacological studies and modeling network dysfunctions.

## Introduction

Human pluripotent stem cell (hPSC)-derived neurons possess great promise for unraveling the mechanisms of diseases with genetic susceptibility^1^. They also provide a human-specific model for *in vitro* research in neurotoxicology and drug discovery with the potential of reducing the use of animal studies^2^. Multiple differentiation protocols have been developed for hPSC-derived cortical neurons, all of which aim at the fast and consistent production of neurons^3–6^. Several methodological advances have improved the controllability of culture conditions, for example, feeder-free cultures of hPSCs and defined mediums devoid of serum^7,8^. Additionally, an efficient neural induction of the default anterior central nervous system (CNS) phenotype has been achieved with a simple dual SMAD inhibition method by blocking the TGF-β and BMP signaling pathways with small molecules^9^. More recently, defined culture matrices such as human recombinant extracellular matrix (ECM) proteins have emerged as potent alternatives for mouse laminin or Matrigel, which are animal-derived products known to suffer from batch-to-batch variation^10–12^. Defined ECM molecules are used both in culture of hPSCs and in neural differentiation and have even been shown to improve the functional maturation of neurons^10–13^. These improvements, among others, are essential for making neural differentiation methods more controllable and comparable between different laboratories and pave the way for more reliable findings in the application of hPSC-derived neurons.

The emergence of neuronal network activity during development is important in regulating neuronal migration, differentiation and apoptosis^14–16^. During CNS development, including that of the cerebral cortex, network formation starts as the newly born neurons elongate their axonal projections, find paths and form synaptic connections^16^. After the initial formation of excessive functional connections, the developing networks undergo refinement of some connections and strengthening of others^16^. Large populations of neurons generate synchronous activity that has a central role in fine-tuning connectivity during CNS development^16,17^. These synchronous oscillations have been described in the cerebral cortex of premature human fetuses at approximately 20 weeks of gestation^18,19^ and in the mouse and rat cortexes at birth (P0)^20,21^. Wide-ranging oscillatory activities in the cortex dominate throughout development, ensuring neuronal survival and maturation^22,23^, and in adults, they are believed to be involved in cognitive and perceptual functions and motor actions^24^. The cerebral cortex and its functional dynamics are also affected in many neurological disorders^25–27^. hPSC-derived neurons have been shown to develop membrane properties similar to native neurons *in vivo* and have been reported to respond to pharmacological manipulation and electrical stimulation, as expected from their *in vivo* counterparts^28–30^. Although network-level events are central in both healthy and pathological states, modeling of network-level activity with hPSC-derived neurons is still less described in the literature^31–34^.

Microelectrode arrays (MEA) have been used to measure the connectivity and network activity in neuronal cultures, brain slices and even in awake animals^28,35–37^. A clear advantage of MEAs is that they measure the activity from a population of neurons simultaneously, providing information about network events. They also enable repeated measurements of the same networks over time, allowing follow-up of developmental events or, for example, long-term drug responses^38^. For *in vitro* research, multiwell-format MEAs also facilitate higher throughput analyses^13,39^. These features make MEAs a potential tool for disease modeling, drug screening and toxicology using hPSC-derived neurons. So far, most MEA culture and signal analysis protocols have been established with rodent networks, which are often considered “gold standards” in the field^40,41^. However, direct transfer and validation of these methods to hPSC-derived networks has not been straightforward^28,42–44^.

Here, we report that the differentiation of hPSC-derived cortical cultures on defined human recombinant laminin-521 (LN521) substrates is repeatable and results in the generation of cortical neurons, which form functionally active networks. We utilize MEA technology to characterize the development of hPSC-derived neuronal network activity and compare it with the activity of rat embryonic cortical networks *in vitro*. The results confirm that spontaneous activity arises through similar developmental stages and on comparable time scales in both hPSC-derived and rat cortical networks. Both network types show typical synchronous activity; however, hPSC-derived networks present unique characteristics in burst patterns that distinguish them from their rat counterparts. The present data validate hPSC-derived neuronal networks as a reliable model for cortical activity development and consolidate their utilization in applications such as disease modeling, where network malfunctions are considered key characteristics.

## Results

### Generation of neural progenitor cells on human recombinant LN521 substrate

**Figure 1:**
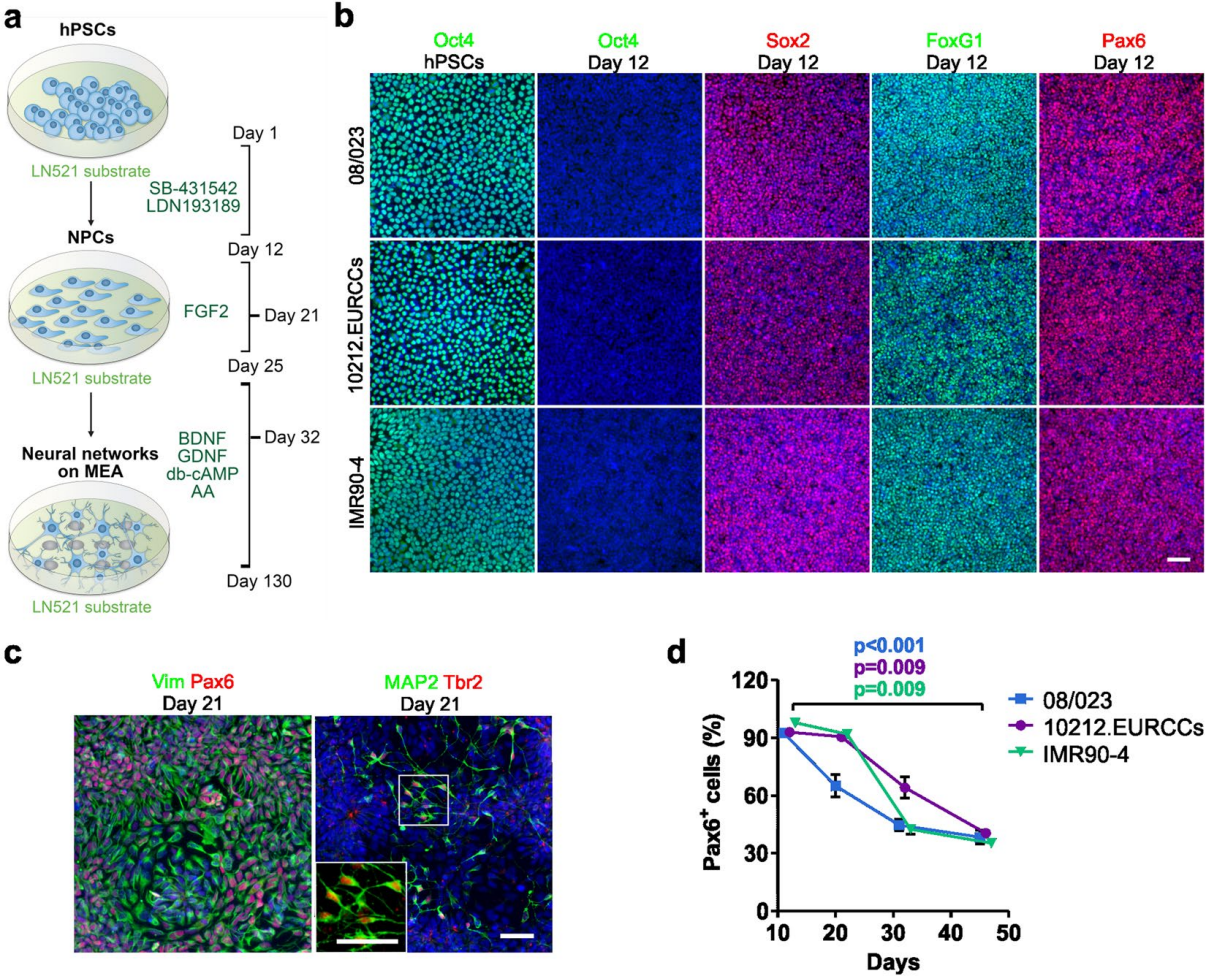
Neural induction of hPSCs. (**a)** Both hPSC culture and neural differentiation were performed on LN521 substrate. Cortical neurons were induced with dual SMAD inhibition, expanded in the presence of FGF2, and matured with support by a selection of neurotrophic factors. Neural progenitor cells (NPCs) could be cryopreserved at day 21 and plated at day 32 for final experiments, including microelectrode array (MEA) measurements. **(b)** Three different hPSC lines (one hESC line, 08/023 and two hiPSC lines, 10212.EURCCs and IMR90-4) were characterized for their efficiency in producing neuroectodermal cells in response to 12-day neural induction by dual SMAD inhibition. Cells were stained for pluripotency marker Oct4 at the pluripotent stage and after 12 days of neural induction. The presence of early neuroectodermal markers was evaluated with Sox2, FoxG1 and Pax6 staining. **(c)** After 21 days of differentiation, the culture contained vimentin- and Pax6-positive NPCs that could be cryopreserved. Additionally, the first Tbr2- and MAP2-positive neurons were detected at this time point. **(d)** The percentages of Pax6-positive cells were quantified at four time points of differentiation (mean ± s.e.m., n = 5–14, data derived from 1–3 independent differentiations). Statistical analysis was performed with the Mann-Whitney U test to compare differences between day 12 and day 46 within each hPSC line, and significant p-values are presented in the image. The scale bar is 50 µm in all images.

To achieve controlled, repeatable protocol producing cortical neurons, we utilized and modified previously described methods for culturing hPSCs and differentiation of neurons that can form spontaneously active networks^6,11^ (Fig. 1a). The hPSCs were cultured in feeder-free conditions using LN521 substrate and Essential 8 (E8) medium according to the protocol from Hongisto *et al*. (2017). The neural differentiation protocol was modified from Shi *et al*. (2012) and further optimized for the LN521 substrate to achieve more defined culture conditions and to support the functional development of neuronal networks, as we have previously described^13^.

To assess the repeatability of the optimized culture protocol, experiments were performed with one human embryonic stem cell (hESC) line (08/023) and two human induced pluripotent stem cell (hiPSC) lines (10212.EURCCs and IMR90-4). All hPSC lines expressed the pluripotency marker Oct4 when grown as a monolayer culture on LN521 substrate in E8 medium (Fig. 1b). After a 12-day neural induction stage with dual SMAD inhibition, the pluripotency marker Oct4 was downregulated, and homogenous expression of neuroectodermal markers Sox2 and Pax6 was apparent, indicating efficient neural conversion (Fig. 1b). In addition, the anterior cortical fate of the cells was identified by FoxG1 staining (Fig. 1b).

After the neural induction stage, vimentin- and Pax6-positive neural stem and progenitor cells (now referred to as NPCs) could be efficiently expanded and differentiated in FGF2-containing media (Fig. 1a,c, Supplementary Fig. S1). Differentiation induced the emergence of first Tbr2- and MAP2-positive cortical neurons (Fig. 1c). Furthermore, NPCs could be cryopreserved at day 21 to create master cell banks. As a comparison to LN521 substrate, we also tested neural induction on the commonly used Matrigel matrix. Cells cultured on Matrigel matrix showed similar homogenous Pax6 staining, and the subsequent differentiation on mouse laminin generated FoxG1- and vimentin-positive NPCs (Supplementary Fig. S2).

After thawing, the NPCs were expanded until day 32 and then used for experiments (Fig. 1a). Neuronal maturation was promoted in the presence of neurotrophic factors BDNF, GDNF, cAMP and ascorbic acid. The number of Pax6-positive NPCs gradually decreased from 93–98% at day 12 to 35–41% at day 46 in all studied hPSC lines, suggesting neuronal maturation (08/023, p < 0.001; 10212.EURCCs, p = 0.009; and IMR90-4, p = 0.009; Fig. 1d). Taken together, neural induction and expansion of NPCs on LN521 substrate was efficient, and the produced cells represented an anterior cortical phenotype.

### Development of cortical layer-specific neurons

**Figure 2:**
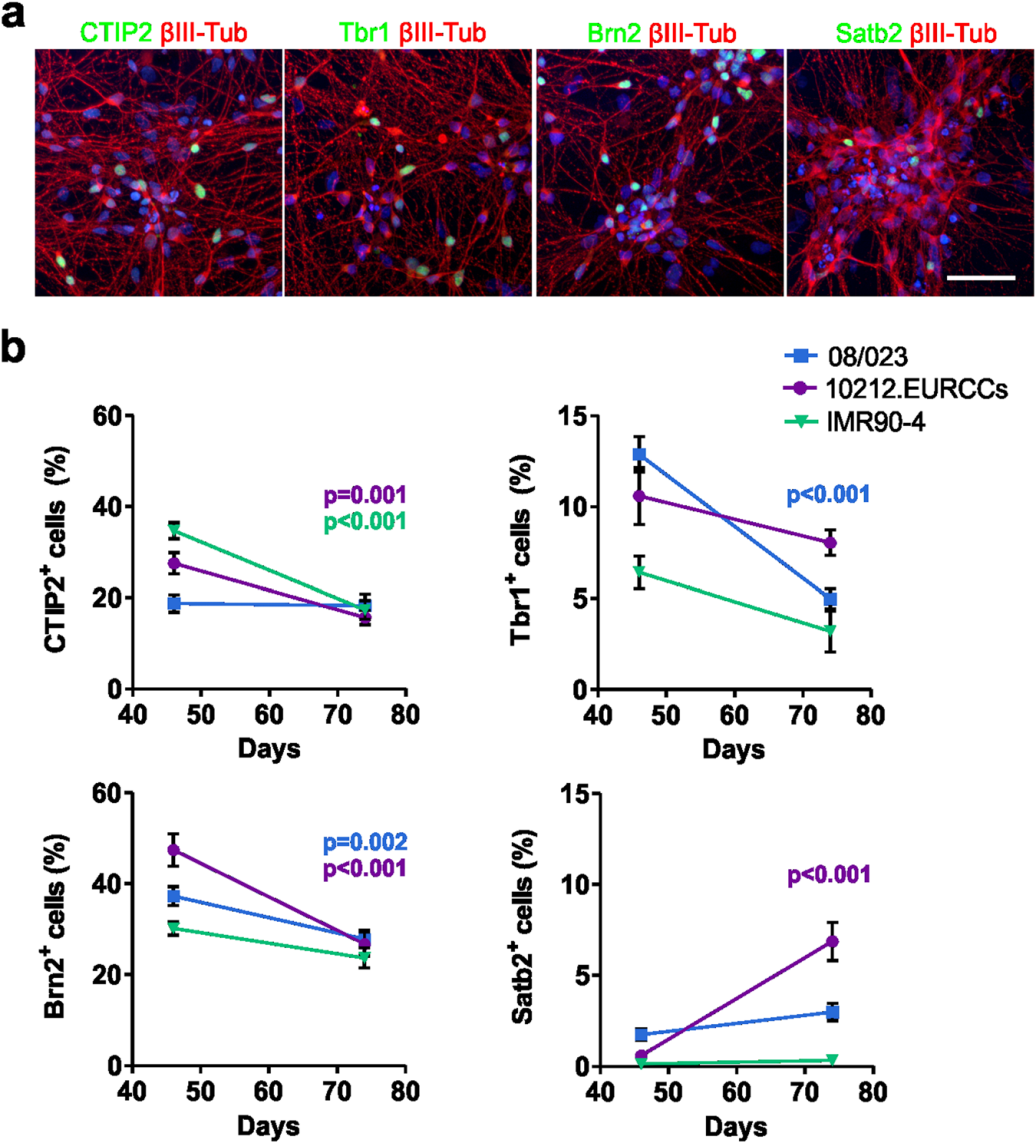
Development of cortical layer-specific neurons. (**a)** Immunocytochemical staining verified the presence of cortical layer-specific neurons expressing the early-born deep layer markers CTIP2 and Tbr1 and the later-born upper layer markers Brn2 and Satb2. Images are representative of the 08/023 hPSC line. Scale bar is 50 µm. **(b)** The number of positive cells for cortical layer-specific markers was quantified from neuronal cultures differentiated from all three hPSC lines at days 46 and 74 of differentiation, and the data are presented as the mean ± s.e.m. (for each hPSC line n = 5–51, data from 1–3 independent differentiations). Mann-Whitney U test was performed to determine statistically significant differences in time within each hPSC line. Significant p-values are presented in the images.

Neurogenesis produced neurons specific for both deep and upper cortical layers. The early-born deep layer neurons were identified by immunocytochemical staining of transcription factors COUP-TF-interacting protein 2 (CTIP2) and T-box homeobox protein 1 (Tbr1) (Fig. 2a, Supplementary Fig. S3a), both having similar temporal expression patterns in the studied hPSC lines. Higher CTIP2 expression, 19–35%, was detected at day 46 and decreased thereafter to 16–18% by day 74 (08/023, p = 0.802; 10212.EURCCs, p = 0.001; and IMR90-4, p < 0.001; Fig. 2b). Similarly, Tbr1 expression showed a minor decrease from 6–13% to 3–8% from day 46 to day 74 (08/023, p < 0.001; 10212.EURCCs p = 0.117, and IMR90–4, p = 0.066; Fig. 2b). Additionally, later-born upper layer neurons positive for POU domain transcription factor Brn2 (also known as POU3F2) and special AT-rich sequence-binding protein 2 (Satb2) were detected in the cultures (Fig. 2a, Supplementary Fig. S3a). The Brn2-positive neurons appeared first in all hPSC lines with higher expression at day 46, 30–47%, followed by a decrease to 24–27% at day 74 (08/023, p = 0.002; 10212.EURCCs, p < 0.001; and IMR90-4, p = 0.050; Fig. 2b). The number of Satb2-positive late-born neurons was low at the studied time points (Fig. 2b). A very modest increase was detected, ranging from 0–2% to 0–7% between days 46 and 74 (08/023, p = 0.052; 10212.EURCCs, p < 0.001; and IMR90-4, p = 0.371; Fig. 2b). In conclusion, the differentiation protocol produced both deep and upper layer cortical neurons in a similar manner for all studied hPSC lines.

### Maturation of hPSC-derived and rat embryonic cortical cultures

**Figure 3:**
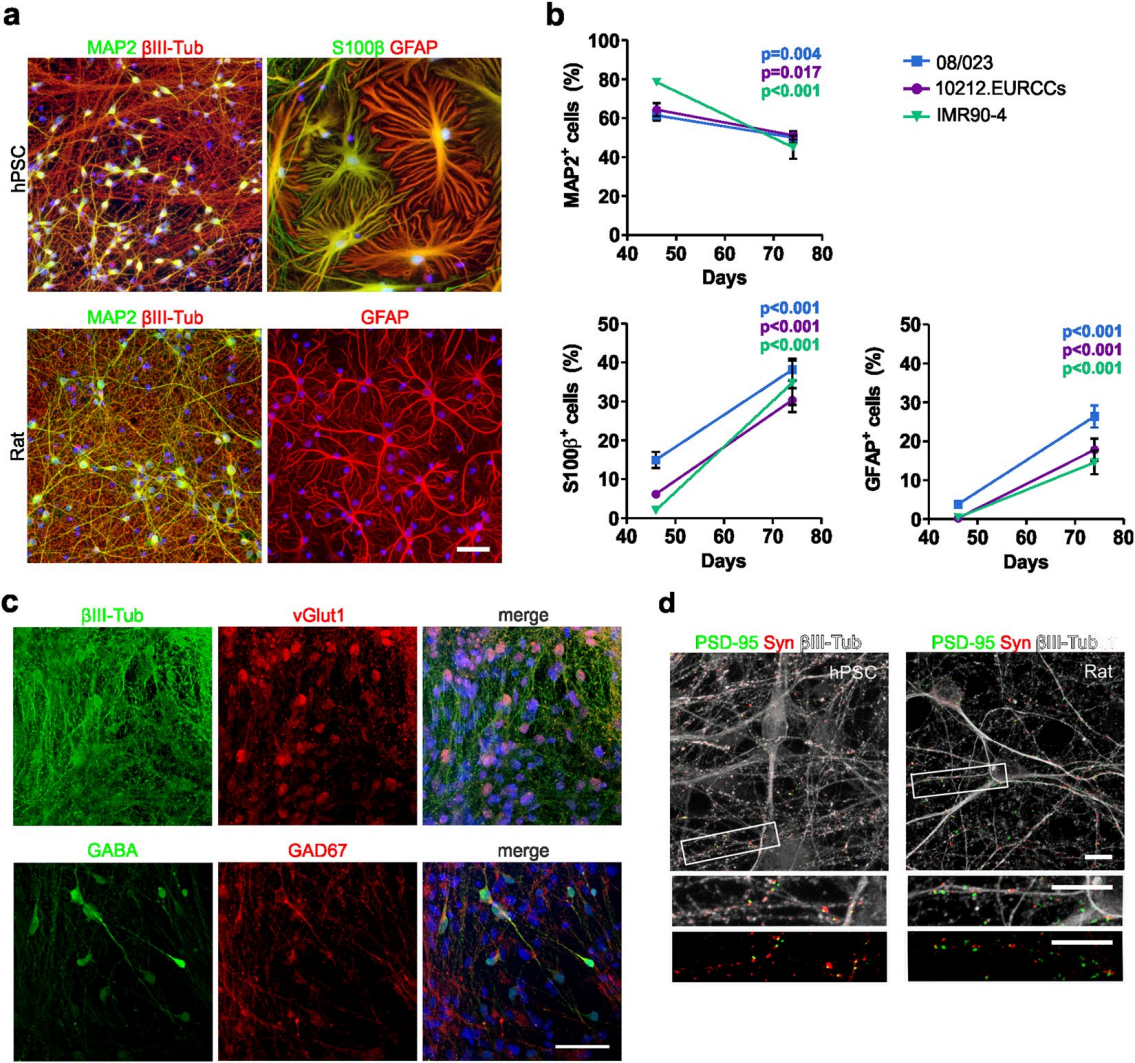
Maturation of hPSC-derived and rat embryonic cortical cultures. (**a)** Immunocytochemical staining of neurons (MAP2 and βIII-tubulin) and astrocytes (GFAP and S100β) in hPSC-derived (hPSC line 08/023) and rat primary cultures. Dapi nuclear staining is shown in blue. Scale bar represents 50 µm. **(b)** The number of neurons and astrocytes was quantified from cultures differentiated from all three hPSC lines at days 46 and 74. Quantitative data are presented as the mean ± s.e.m. (MAP2 n = 7–42, S100β and GFAP n = 9–44, data derived from 1–3 independent differentiations). The Mann-Whitney U test was performed between time points within each hPSC line, and significant p-values are presented in the images. **(c)** The hPSC-derived neuronal cultures consisted of vGlut1-positive glutamatergic and GABA- and GAD67-positive GABAergic neurons. Dapi nuclear staining is shown in blue, and the scale bar represents 50 µm. **(d)** Both hPSC-derived (day 61) and rat embryonic (day 22) cortical cultures formed networks with excitatory synapses positive for synaptophysin (Syn) and PSD-95. Insets show higher magnification, and the scale bars are 10 µm in all images.

Next, the cell type composition of the hPSC-derived neural cultures differentiated on LN521 substrate was characterized by MAP2 and βIII-tubulin staining for neurons and S100β and GFAP staining for astrocytes (Fig. 3a). For comparison studies on the MEAs, the rat embryonic cortical cells were also cultured and characterized with the same markers (Fig. 3a).

The number of neurons and astrocytes was quantified from hPSC-derived cortical cultures. The percentage of MAP2-positive neurons was 61–79% at day 46 and decreased significantly to 45–51% at day 74 in the studied hPSC lines (08/023, p = 0.004; 10212.EURCCs, p = 0.017, and IMR90-4; p < 0.001; Fig. 3b). In contrast, the number of S100β- and GFAP-positive astrocytes increased in a temporal manner, suggesting subsequent astrogenesis from the common neural progenitor pool. The percentage of S100β-positive astrocytes increased significantly from 2–15% to 30–38% between day 46 and day 74 in the studied hPSC lines (08/023, p < 0.001; 10212.EURCCs, p < 0.001; and IMR90-4, p < 0.001; Fig. 3b). GFAP expression appeared later, as only 0–4% GFAP-positive astrocytes were detected at day 46, while later, at day 74, their number was significantly increased to 15–26% (08/023, p < 0.001; 10212.EURCCs, p < 0.001; and IMR90-4, p < 0.001; Fig. 3b). Interestingly, almost all GFAP-expressing astrocytes were also S100β-positive (Supplementary Fig. S3b). The neuronal population was further characterized as consisting of both vGlut1-expressing glutamatergic neurons and GABA- and GAD67-positive GABAergic neurons (Fig. 3c). Importantly, both hPSC-derived and rat embryonic cortical neurons formed structural synapses identified with juxtaposed synaptophysin and PSD-95 puncta (Fig. 3d). In summary, both rat and hPSC-derived cortical neurons established synaptically connected networks that were intermingled with astroglia.

### Development of spontaneous activity in hPSC-derived and rat cortical networks

**Figure 4:**
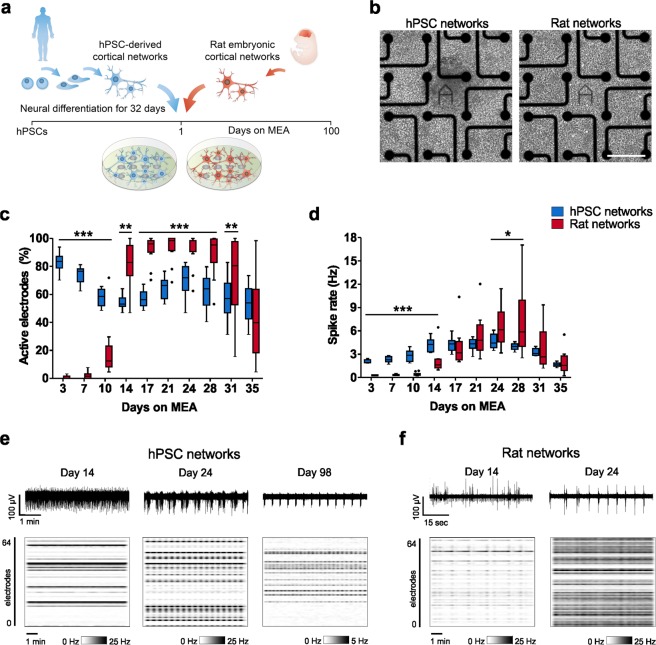
Developmental changes in the spontaneous activity of hPSC-derived and rat cortical networks on MEA. (**a)** hPSC-derived cortical neurons were differentiated for 32 days before plating on MEAs. Rat cortical neurons were dissected from E17-E18 embryos and directly plated on MEAs. **(b)** Phase contrast images showing hPSC-derived and rat networks on a MEA. The scale bar is 200 µm. **(c)** Percentage of active electrodes per MEA well in hPSC-derived and rat networks over time. Electrodes detecting >10 spikes/min were considered active. **(d)** Development of **s**pike rate (Hz) per MEA well in hPSC-derived and rat networks over time. hPSC network data are from line 08/023. Both hPSC and rat network data consist of n = 12 networks per group. Data are presented as Tukey box plots. Mann-Whitney U test was performed to compare differences between hPSC-derived and rat networks, and significance within each time point is denoted in the images as *p < 0.05, **p < 0.01 and ***p < 0.001. **(e)** Representative images of the spontaneous activity recorded from single electrodes of hPSC-derived networks over 5 minutes and **(f)** from rat networks over 1 minute at different time points. Raster plots below show the intensity of spike activity on a MEA during a 10 min recording.

To study the spontaneous activity of hPSC-derived cortical networks differentiated on LN521 substrate and compare their functional properties to the widely used rat embryonic cortical networks, we followed network development with regular MEA measurements over 100 days. The hPSC-derived cortical cells were plated on MEAs at day 32 of differentiation (now called day 1 on MEA), and primary rat cortical neurons (from E17-E18 embryos) were plated directly on MEAs (Fig. 4a). Cell density has been shown to be critical for the timeframe of activity development on MEAs^34,45^, and therefore, cells were plated at high densities (Fig. 4b). Measurements performed on 64-electrode MEAs (8 × 8 grid) revealed that this facilitated the detection of activity from hPSC-derived networks soon after plating (Fig. 4c). The hPSC-derived networks expressed widespread activity from day 3 on MEA onwards, and the percentage of active electrodes (>10 spikes/min) ranged from 53% to 84% over 35 days on MEA. The rat networks showed latency in the onset of activity development (Fig. 4c). In the rat networks, the number of active electrodes was 2% at day 3 on MEA, reaching almost 100% on day 21 and then declining towards day 35.

The median spike rate per well increased early in the hPSC-derived networks, starting from day 3 and through day 14 on MEA (Fig. 4d). However, the rat networks showed higher spike rates during the most active state at days 24 to 28 on MEA (Fig. 4d). Here, the peak activity for both groups was detected after 24 days on MEA (hPSC median 4.4 Hz and rat median 6.1 Hz, Fig. 4d). After 35 days, the rat networks experienced a typical terminal decline in activity, while hPSC-derived networks settled on a plateau (Fig. 4d,e). We also looked at activity development in hPSC-derived neurons differentiated on the commonly used mouse laminin substrate (Supplementary Fig. S4). Similar to our earlier reports^13^, the activity development of hPSC-derived neurons was more efficient on LN521 substrate compared to mouse laminin, as indicated by both the number of active electrodes and spike rate in the networks.

Representative images of firing patterns detected with single electrodes and entire MEA arrays revealed the developmental organization of uncorrelated spike trains into highly synchronous bursts in both hPSC-derived and rat networks from day 14 to 24 on MEA (Fig. 4e,f). In hPSC-derived networks, this synchronous bursting was still apparent after 98 days on MEA (Fig. 4e). Taken together, hPSC-derived networks differentiated on LN521 substrate develop activity across the MEA array in a comparable time period to the rat networks. They can reach spike rates close to that of the rat counterparts and maintain activity for almost 100 days on MEA.

### Both hPSC-derived and rat neurons form highly connective networks

**Figure 5:**
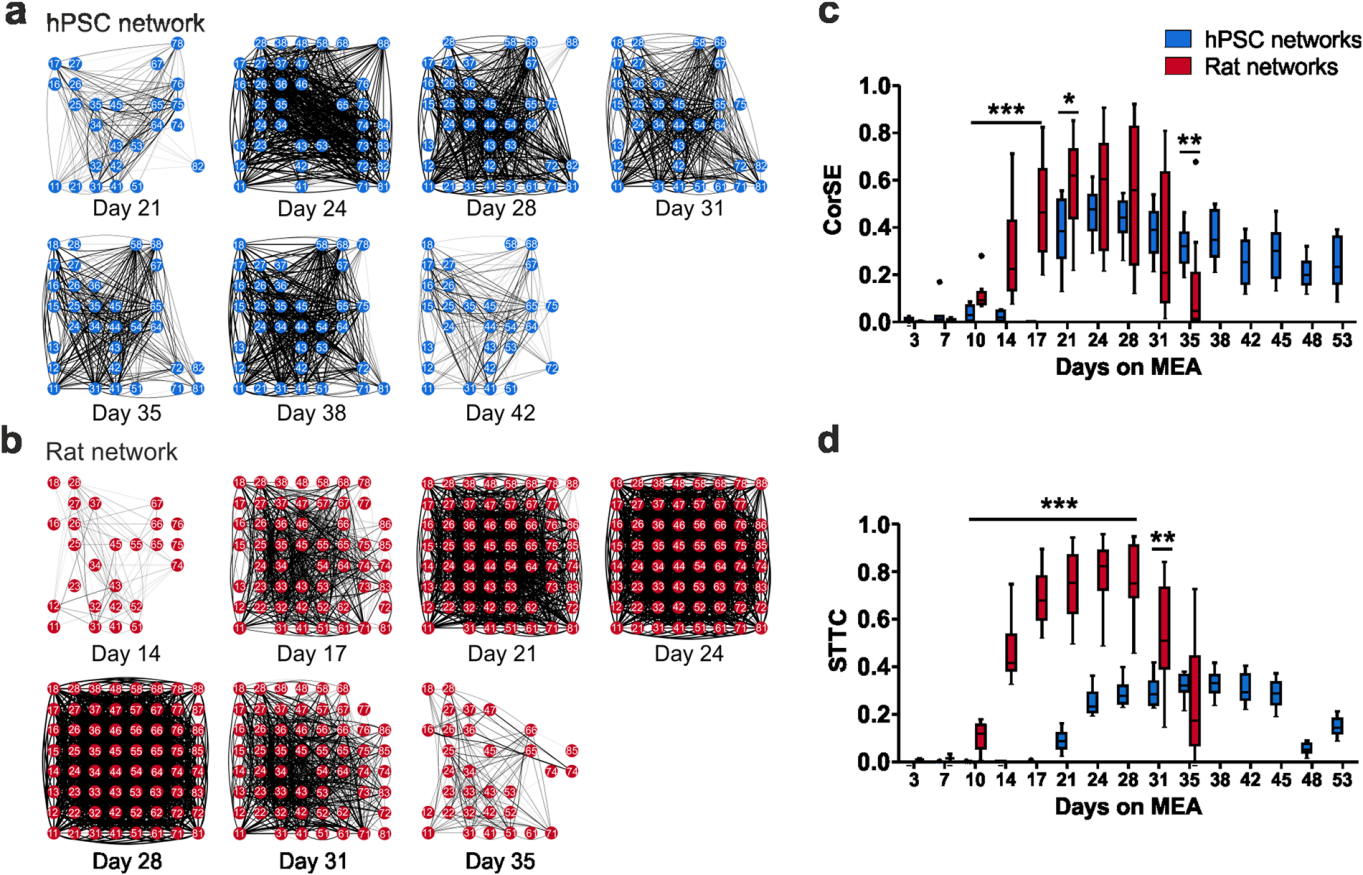
Analysis of functional connectivity development. (**a**) Functional connectivity maps from the hPSC-derived network over 10 minutes of recording between days 21 and 42 on MEA and **(b)** from the rat network between days 14 and 35 on MEA. Electrodes in an 8 × 8 array with functional connections (lines) are presented during the measurement time. An arbitrary connectivity strength (CorSE) value of 0.7 was used as the threshold for plotting. **(c)** Average connectivity strength (CorSE) is calculated from all channel pairs for hPSC-derived and rat networks. **(d)** Network synchronization of hPSC-derived and rat networks described by the spike time tiling coefficient (STTC). Data hPSC data are from line 08/023. Both hPSC and rat network data consist of n = 12 networks per group; data are shown as Tukey box plots. Mann-Whitney U test was performed to compare the two groups at each time point. Statistical significances are marked as *p < 0.05, **p < 0.01 and ***p < 0.001.

Functional connectivity is typically measured as simultaneously occurring events of activity originating from spatially distant areas^46^. To determine how array-wide activity develops in hPSC- and rat-derived networks, we performed connectivity analysis based on the entropy between electrode pairs^47^. Representative connectivity maps showing results from one network of hPSC and rat groups over 10 minutes of recording revealed a gradual increase in functional connections over time (Fig. 5a,b). The first functional connections were observed in hPSC-derived networks after 21 days on MEA and in rat networks after 14 days on MEA (Fig. 5a,b). Connections expanded and strengthened progressively in both hPSC-derived and rat networks (Fig. 5a,b). When connectivity strength (CorSE) values were quantified, a considerably higher level of connectivity was observed in rat networks compared to hPSC-derived networks between 10 and 21 days on MEA (Fig. 5c). The strongest network connectivity was detected after 24 days on MEA (hPSC median CorSE 0.48 and rat median CorSE 0.60). While rat networks were silenced after 35 days on MEA, in hPSC-derived networks, the connectivity persisted between certain areas of the networks over several days (Fig. 5a–c). We further complemented the analysis by calculating the spike time tiling coefficient (STTC), which measures network synchronization in pairs of electrodes^48^. The results from STTC analysis confirmed the earlier observations with CorSE analysis, showing that although both hPSC-derived and rat networks form connections across the culture, in the rodent data, the synchronous activity was initiated earlier and involved a larger fraction of the network (Fig. 5d).

### Bursting behavior differs between hPSC-derived and rat networks

**Figure 6:**
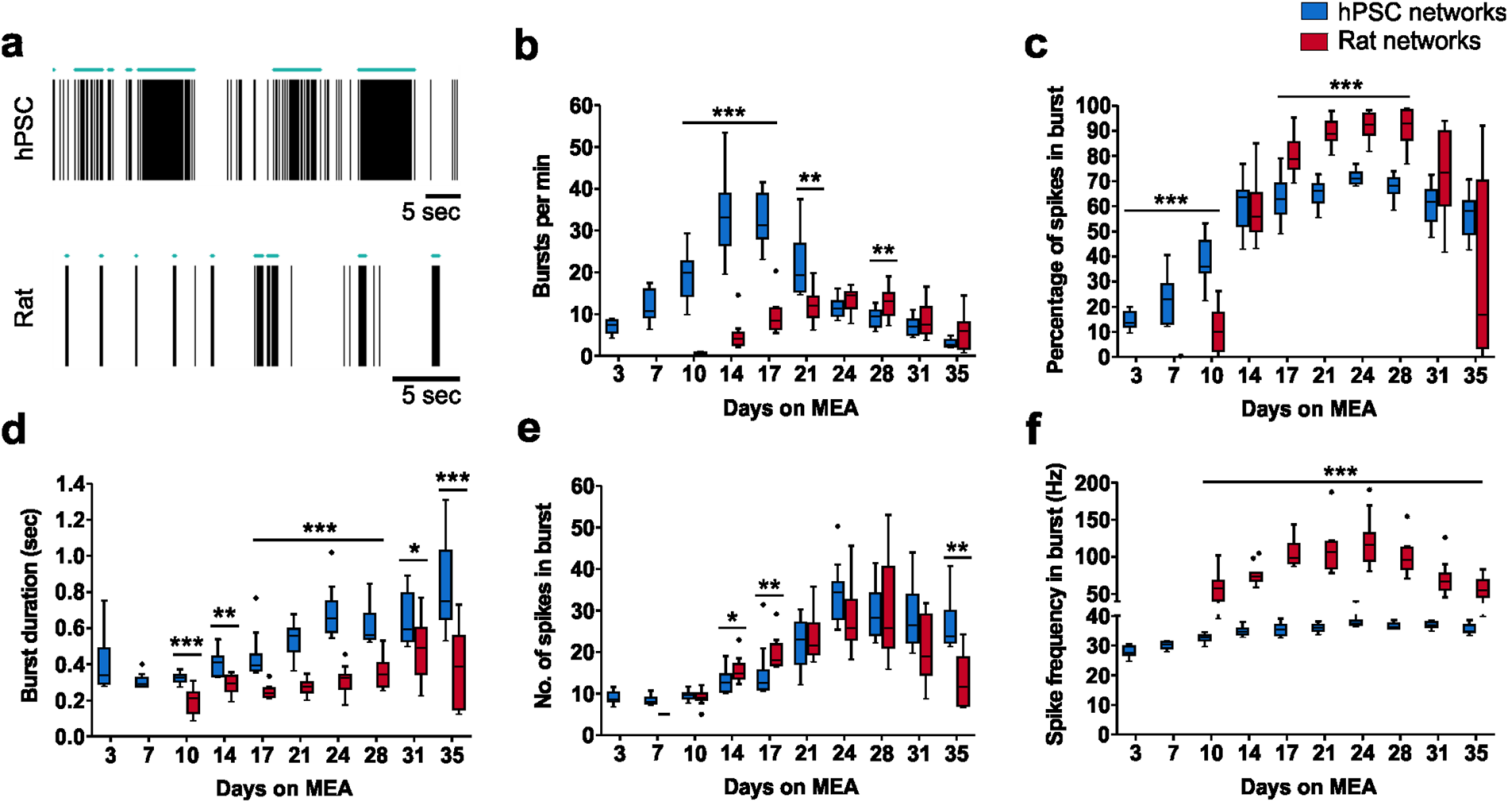
Burst features in hPSC-derived and rat cortical networks. (**a**) Raster plots showing typical spike and burst activity of one electrode from both hPSC-derived and rat data at day 24 on MEA. The green horizontal lines denote detected bursts. Temporal changes in burst features including **(b)** number of bursts per minute, **(c)** percentage of spikes in burst, **(d)** burst duration, **(e)** number of spikes in bursts, and **(f)** spike frequency in bursts. Data consist of n = 12 networks for both hPSC-derived (hPSC line 08/023) and rat networks and are presented as Tukey box plots. Mann-Whitney U test was performed to compare differences between the two groups at each time point, and significance is denoted as *p < 0.05, **p < 0.01 and ***p < 0.001.

Spontaneous bursts arise during early activity development in the cerebral cortex and are important for neuronal circuit formation^16^. The tonic firing detected soon after cell plating gradually transformed into bursts with a rich repertoire of patterns, which could be reliably detected from both hPSC-derived and rat neuronal signals (Fig. 6a). The number of bursts and the percentage of spikes participating in the bursts progressively increased in both network types (Fig. 6b,c). During the peak of activity, the percentage of spikes recruited into bursts was generally higher in rat networks than in hPSC-derived networks, which also presented substantial spiking between condensed bursts (Fig. 6a,c). The hPSC-derived networks initially fired short bursts at a high frequency (Fig. 6b,d). When the well median values were examined at day 14, the burst frequency was 33 bursts per min, the burst duration was 0.4 sec, and bursts contained fewer than 13 spikes (Fig. 6b,d,e). Once networks matured, less frequently occurring and longer-duration bursts became the more dominant form of activity (Fig. 6a,b,d). During the peak of activity at day 24, the burst frequency was 11 bursts per min, the burst duration was 0.7 sec, and bursts contained 34 spikes (Fig. 6b,d,e). In the rat networks, the generation of bursting activity was steady, and the highest number of bursts, 15 per min, was detected during the peak of activity on day 24 (Fig. 6b). At that point, the median burst duration was 0.3 sec (Fig. 6d). Thus, hPSC-derived networks expressed longer bursts than the rat networks. Although rat bursts consisted of a similar number of spikes as observed in hPSC-derived networks (Fig. 6e), the spike frequencies in bursts were different across all time points (Fig. 6f). The median spike frequency in bursts was below 40 Hz in the hPSC-derived networks, whereas in rat networks, up to 100 Hz was observed during the peak of activity (Fig. 6f). Taken together, the hPSC-derived and rat networks express bursts that differ mostly in four properties: temporal development of bursts, burst duration, percentage of spikes recruited in bursts and spike frequency inside bursts.

### hPSC-derived and rat networks show similar pharmacological responses

**Figure 7:**
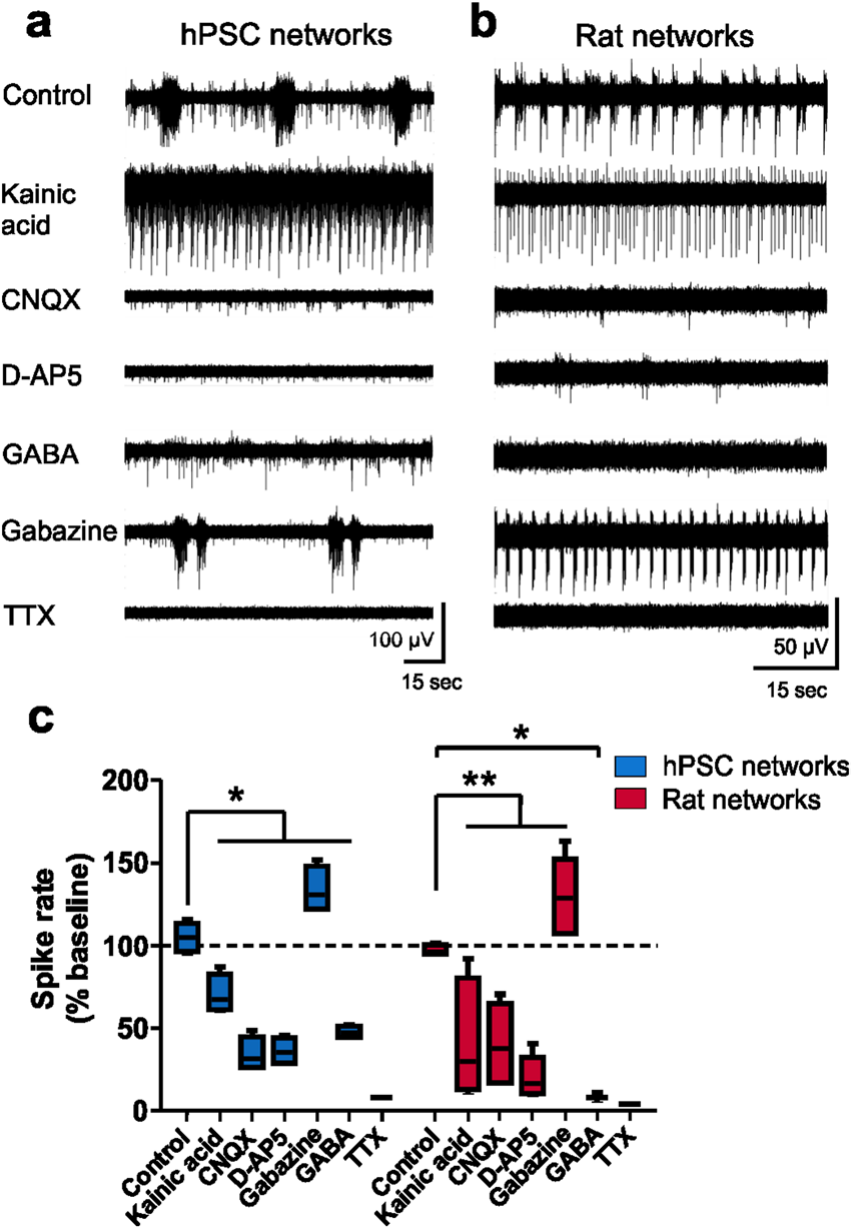
Pharmacological responses of hPSC-derived and rat cortical networks. (**a)** Typical activity patterns in response to different pharmacological treatments recorded from single electrodes of hPSC-derived networks at 29 days on MEA over 2 minutes and **(b)** from rat networks at 22 days on MEA over 1 minute. **(c)** Percentage change in spike rate compared to baseline measurement from the same well. Data consist of n = 4 networks for hPSC networks (hPSC line 08/023) and n = 7 for rat networks and are presented as a Tukey box plots. Mann-Whitney U test was performed to compare differences between the treatment groups, and significance is marked as *p < 0.05, **p < 0.01 and ***p < 0.001.

Next, we evaluated whether the observed network activity was synaptically driven and whether we could pharmacologically manipulate activity in both network types. Experiments were carried out once synchronous activity was well established after 29 days on MEA for hPSC-derived networks and after 22 days on MEA for rat networks. Typical firing patterns showed that common glutamatergic and GABAergic agonists and antagonists evoked similar responses in both hPSC-derived and rat networks (Fig. 7a,b). The glutamatergic agonist kainic acid reduced the spiking activity and disorganized the synchronous network bursts down to tonic spike trains in both groups similarly (Fig. 7a–c), as observed earlier^49^. A concentration-dependent response to kainic acid was also observed with the rat networks (Supplementary Fig. S5). In both network types, the glutamatergic AMPA/kainate antagonist CNQX and the NMDA antagonist D-AP5 both efficiently reduced activity, verifying that the synchronous network events involve synaptic inputs (Fig. 7a–c).

The presence of the inhibitory system is not often observed in hPSC-derived forebrain cortical cultures^50^, and therefore, we tested the effect of the GABA and GABA_A_ receptor antagonist gabazine on network activity (Fig. 7a,b). GABA suppressed the excitatory activity in both groups but more efficiently in the rat networks (Fig. 7c). Gabazine blocked GABAergic signaling in both rat and hPSC-derived networks, leading to increased spike rates (Fig. 7c). Finally, we confirmed that the activity recorded with MEAs originated from neuronal excitation, since all activity was silenced with the voltage-dependent sodium channel blocker tetrodotoxin (TTX, Fig. 7a–c). In summary, both hPSC-derived and rat networks presented network activity that was sensitive to synaptic modulators, and most importantly, the networks contained a functional inhibitory system.

### Principal component analysis of the features of MEA activity distinguishes hPSC- and rat-derived networks

**Figure 8:**
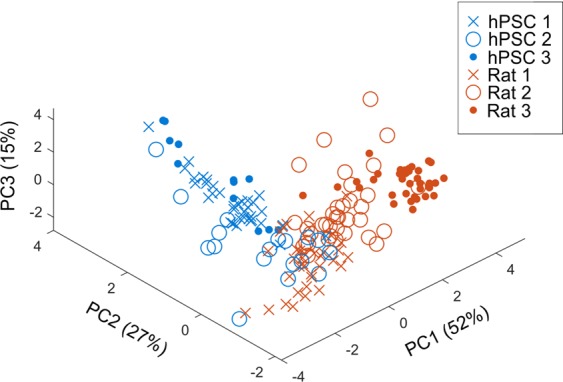
Principal component analysis of features of MEA activity of hPSC-derived and rat datasets. Principal component analysis (PCA) was performed to cluster hPSC-derived and rat MEA data based on seven activity features. Analysis was performed on three separate hPSC-derived (hPSC 1–3) and rat data sets (Rat 1–3) that all included 4–12 networks. Data were analyzed from time points showing the highest spike rates during development and two of their neighboring measurement time points. Data are plotted in 3-dimensional space, where each of the principal components accounts for the amount of variation of the data shown in parenthesis. Each data point in the scatter plot represents one network at a particular time point.

Principal component analysis (PCA) can efficiently cluster MEA data based on activity feature profiles and has been used for studying the properties of various brain region-specific cells and the effects of chemical exposure^40,51^. Therefore, we applied PCA to obtain conclusive results from the functional comparison between the hPSC-derived and rat networks. Both network types may have variability in the onset of activity and synchrony; therefore, analysis was performed on three separate experiments at the time point showing the highest spike rate and two of its neighboring measurement days (Supplementary Figs. S6 and S7). To measure the differences, we used several spike, burst and synchrony features. A clear segregation of the hPSC-derived and rat recordings was observed when the 7-dimensional feature vectors were plotted onto the first three principal components (Fig. 8). The first principal component (PC1) accounted for more than half of the variation (52%) between the two networks, and PC2 and PC3 explained 27% and 14% of the variation, respectively. However, there was a slight overlap between the second hPSC batch (hPSC 2) and the first rat batch (Rat 1), suggesting that single datasets cannot be reliably used to discriminate between two types of recordings. In fact, in the rat networks group, internal variation among the three individual experiments was well explained by the first PC. The hPSC-derived networks were better explained by the second PC, and although variability within experiments was observed, a clear segregation between the three independent hPSC experiments (hPSC 1-hPSC 3) was not evident. In conclusion, PCA was used as a promising tool for summarizing multiple activity features from different MEA experiments. It successfully displayed differences in the individual MEA recordings and identified two separate clusters for hPSC-derived and rat networks suggestive of their distinct functional characteristics.

## Discussion

We established here hPSC-derived cortical networks using highly defined culture methods resulting in consistency of differentiation and distinct functional properties. We have previously shown improved functionality of neurosphere-differentiated networks on MEA with laminin alpha5 substrates^13^. We now apply LN521 substrate for hPSC culture^11^ and the subsequent adherent neuronal differentiation, and for the first time, we show a detailed network-level functional comparison between hPSC-derived and rat embryonic cortical cultures. The results demonstrate that hPSC-derived cortical networks present unique activity patterns, but the stages of development over time reflect those observed in rat cortical networks. Thus, these results validate the hPSC-derived networks as a representative model for cortical activity development *in vitro*.

Over the years, increasing knowledge of the key signaling molecules involved in neurodevelopment has been utilized for the improvement of neuronal differentiation methods^9,52^. Less attention has been drawn to the ECM components, although they are known to mediate cellular effects through signaling via membrane receptors^53,54^. Laminins are a major class of ECM molecules and are essential during embryogenesis and neural development, regulating neuronal proliferation, differentiation and migration^53,55^. The human recombinant laminin isoform LN521 has been shown to support the pluripotency of hPSCs in feeder-free culture^7,11,12^ and has been applied to dopaminergic differentiation of multiple hESC and hiPSC lines^10,56^. We have also demonstrated that laminin α5 substrates, including LN521, improve the functional activity of hPSC-derived neuronal networks on MEA^13^. Therefore, here LN521 was chosen as the sole substrate for maintaining the undifferentiated hPSCs^11^ and for supporting efficient neural induction, cortical differentiation and functional development. Using optimized methods, all three of the studied hPSC lines presented over 90% neural conversion as shown by Pax6 staining after dual SMAD inhibition, and the generated NPCs expressed typical markers of the forebrain cortical phenotype. Importantly, the NPCs were cryopreservable, and with further maturation, they readily differentiated into mature neural cell types, neurons and astrocytes. The neuron population showed typical temporal profiles of cortical layer marker expression, in which the deep layer neurons were produced first followed by generation of upper layer neurons, similar to previous reports^6^. The majority of the neurons consisted of glutamatergic projection neurons and GABAergic interneurons, and the formation of structurally mature synapses was observed.

Both neurons and astrocytes are derived from the same neuroepithelial pool in a distinct temporal order with neurogenesis preceding astrogenesis^57^. Here, the glial switch was apparent during differentiation, as all hPSC lines produced first MAP2-positive neurons followed by an increased expression of S100β and GFAP, phenotypical markers for astrocyte progenitors and mature astrocytes, respectively. After 74 days of differentiation, over 30% of the cell population consisted of S100β-expressing astrocytes, of which at least half were double-positive for GFAP, while neurons accounted for approximately 50% of the cells in the culture. Many of the previous cortical differentiation protocols have described lower amounts of astrocytes (<10%), even after 80–100 days of differentiation^4,6,58^. Astrocytes are known to promote synaptogenesis and functional maturation of neurons and are also important for the synchronization of networks^59,60^. Consequently, hPSC-derived neurons are often cocultured with rodent astrocytes to improve functional activity^29,38^. However, major drawbacks of these cocultures include species differences and the risk of masking the phenotypes of hiPSC-derived neurons, which is particularly critical for studies focused on disease modeling^61^. Attempts have been made to overcome the species differences by replacing rodent cells with hPSC-derived astrocytes^31,62^. We and others have previously shown functionally active networks with neurons and astrocytes differentiated through EB or neurosphere aggregate stage^34,63,64^. Here, we also present a humanized, fully adherent culture where astrocytes originate endogenously from the same NPCs as neuronal cells and are able to support the functional development of the networks.

Functional activity arises during early embryonic development and is essential for neuronal survival, migration and differentiation^14,17^. The hPSC-derived neuronal cells are generally considered functionally immature, resembling those of the late embryonic stages in rodents, and indeed, transcriptional profiling has shown similarity between hPSC-derived neural cultures and the cells of mid-gestational human embryos^4,65,66^. Here, we show that the time scale for *in vitro* functional activity development between late-embryonic stage rat cortical networks and hPSC-derived cortical networks predifferentiated for one month are highly similar. Measurements in high-throughput multiwell MEAs demonstrate that activity can be detected from the hPSC-derived networks starting already from the early recording days, and the most active stage can be reached within a comparable time to that of rat cortical networks. Rat cortical cells developed functionally connected networks in 14–21 days on MEA, as similarly described by others^41,42^. Additionally, hPSC-derived neuronal networks formed robust synchrony across the culture as early as 21 days (3 weeks) on MEA. Previous studies have typically reported longer times, up to 20 weeks on MEA, for hPSC-derived networks to reach full maturation^32,38,62^. Furthermore, our data reveal that the hPSC-derived and rat cortical networks undergo similar phases of development in activity patterns. Both networks initially fire uncorrelated single spikes and tonic spike trains, and upon maturation, an increasing connectivity is observed when the activity is orchestrated into highly synchronous network bursts.

Spontaneous bursting is observed in the developing cerebral cortex and is considered important for the formation of neuronal circuits^16^. The hPSC-derived networks initially fired comparatively short bursts at high frequency, but as the networks matured, the bursts became less frequent and longer in duration. Over the recoding period, hPSC-derived cortical networks were characterized by bursts with longer durations and lower spike frequencies compared to rat counterparts. Additionally, they presented a higher number of spikes outside the bursts, all of which may reflect species-specific differences or different states of maturity between the two cell types. In both hPSC-derived and rat cortical networks, activity was synaptically driven, as shown by the inhibition with glutamatergic antagonists and application of GABA. We also confirmed the presence of a functional inhibitory system in both networks, as demonstrated by the increased activity upon pharmacological modulation with a GABA_A_ receptor antagonist. To date, only a few studies have reported robust burst firing of hPSC-derived neuronal cultures^31,34,38,67^, and the activity often arises from purely excitatory glutamatergic networks lacking inhibitory GABAergic inputs^50^.

We demonstrated an extensive, direct *in vitro* comparison between rat cortical networks held as the “gold standard” in the MEA field and human-derived networks, which have been less covered by previous reports^68–71^. PCA analysis based on spike rates, network synchronization and burst features effectively identified differences between the two network types; thus, this approach seems potential for future studies. There is an increasing interest in disease modeling to better understand pathology at the network level and to exploit hPSC-derived neurons in drug screening and neurotoxicological studies^31,72–74^. Validation of methods is of importance^70,74^, as variability in hPSC-derived neuronal culture protocols, functional measurements and analysis procedures may at worst lead to falsely assumed conclusions on disease-specific phenotypes. Standardization also supports evaluations of human and rodent networks for drug screening and toxicological analysis and helps to avoid false negative or positive hits that may compromise further studies.

Based on our results, we report reproducible differentiation of hPSC-derived cortical networks and their stable functional development on MEA. The hPSC-derived networks differ from rat *in vitro* counterparts mostly by their unique bursting properties, whereas the stages of activity development reflect the rodent cortical networks in many ways. Detailed quantification of the functional similarities and differences between the rodent and human *in vitro* networks utilizing the current state-of-the-art methods offers a foundation for future studies involving healthy and pathological networks.

## Methods

### Maintenance of human pluripotent stem cells

The hPSC lines used in this study consist of the in-house derived hESC line Regea 08/023^75^ (total passages 29–31, feeder-free passages 6–8), the in-house derived hiPSC line 10212.EURCCs^76^ (total passages 38, feeder-free passages 6) and the commercial hiPSC line IMR90-4^77^ (WiCell, total passages 46, feeder-free passages 5–9). Lines Regea 08/023 and 10212.EURCCs were derived at the Faculty of Medicine and Health Technology (MET), Tampere University, Finland, which has approval from the Finnish Medicines Agency (FIMEA) for research utilizing human embryos (Dnro 1426/32/300/05) and supportive statements from the regional ethics committee of Pirkanmaa Hospital District for the derivation, culture, and differentiation of hESCs (R05116) and hiPSCs (R08070). Informed consent was obtained from all subjects who provided cell samples. All methods were carried out in accordance with relevant guidelines and regulations. The hPSC lines were maintained on top of a human foreskin fibroblast feeder cell layer in Dulbecco’s modified Eagle’s medium (DMEM) containing 20% KnockOut Serum Replacement (both from Thermo Fisher Scientific) as described previously^78^. Before neural differentiation, hPSCs were transferred and expanded in feeder-free culture on recombinant human laminin-521 (LN521, Biolamina, Sweden) and E8 medium (Thermo Fisher Scientific) according to a previous publication^11^. The pluripotency of hPSC lines was regularly monitored with immunocytochemical staining of Nanog, Oct-3/4, SSEA-3, SSEA-4, TRA-1-81 and TRA-1-60, and the capacity to produce different germ layers in the EB formation assay was verified by staining for α-smooth muscle actin, α-fetoprotein, and Nestin. All cultures maintained normal karyotypes and were mycoplasma free.

### Neural differentiation

The neural differentiation protocol was modified from a previously published method^6^. hPSCs were detached using TrypLE Select (Thermo Fisher Scientific) and plated at a density of 5 × 10^5^ cells/cm^2^ on 100 µg/ml poly-L-ornithine (PO, Sigma) and 15 µg/ml LN521 or Matrigel matrix (Corning)-coated plates in E8 medium containing 10 µM ROCK inhibitor (Y-27632, Sigma). Neural maintenance medium was used as a basal medium and consisted of 1:1 DMEM/F12 with Glutamax and Neurobasal, 0.5% N2, 1% B27 with Retinoic Acid, 0.5 mM GlutaMAX, 0.5% NEEA, 50 µM 2-mercaptoethanol (all from Thermo Fisher Scientific), 2.5 µg/ml Insulin (Sigma) and 0.1% penicillin/streptomycin (Thermo Fisher Scientific). During the neural induction stage (days 1–12, Fig. 1a), the maintenance medium was supplemented with 100 nM LDN193189 and 10 µM SB431542 (both from Sigma), and the medium was changed daily. At day 12, the cells were detached with StemPro Accutase (Thermo Fisher Scientific) and plated at a density of 2.5 × 10^5^ cells/cm^2^ on PO and either LN521 or mouse laminin (Sigma)-coated well plates in neural induction medium containing 10 µM ROCK inhibitor. For neural proliferation (days 13–25), the maintenance medium was supplemented with 20 ng/ml fibroblast growth factor-2 (FGF2, Thermo Fisher Scientific). At days 17, 21 and 25, the neural progenitor cells were passaged with StemPro Accutase and replated in medium containing 10 µM ROCK inhibitor. At day 21, the NPCs were cryopreserved in the same medium containing 10% DMSO (Sigma). For final maturation (days 26–130), the medium was changed to maintenance medium supplemented with 20 ng/ml brain-derived neurotrophic factor (BDNF, R&D Systems), 10 ng/ml glial-derived neurotrophic factor (GDNF, R&D Systems), 500 µM dibutyryl-cyclicAMP (db-cAMP, Sigma) and 200 µM ascorbic acid (AA, Sigma). At day 32, the cells were plated for experiments at a density of 50,000 cells/cm^2^ on plastic well plates or 1 × 10^6^ cells/cm^2^ on microelectrode arrays (MEAs). Plastic well plates were coated with PO and either LN521 or mouse laminin as before and MEAs with 0.1% poly-ethylene-imide (PEI, Sigma) and either 50 µg/ml LN521 or mouse laminin. Medium changes were performed every two to three days.

### Primary rat cultures

Cortex tissue was harvested from embryonic days 17–18 Wistar rat embryos as described previously^79^. Local authority approved the animal license (County Administrative Board of Southern Finland, ESAVI/10300/04.10.07/2016) to conduct the described procedures. All experiments were performed according to institutional guidelines and regulations (University of Helsinki internal license number: KEK17-016). The medium consisted of Neurobasal, 2% B27, 2 mM GlutaMAX and 1% penicillin/streptomycin (Thermo Fisher Scientific). The plating density for the experiments was 100,000 cells/cm^2^ on plastic cell culture wells or 2.5 × 10^5^ cells/cm^2^ on MEAs. Plastic well plates and MEAs were coated with 25 µg/ml poly-D-lysine (PDL, Sigma). The media was changed every two or three days.

### Immunocytochemical staining

Immunocytochemistry was performed as previously described^80^. Primary antibodies consisted of βIII-tubulin (rabbit, 1:2000, GenScript: A01627), βIII-tubulin (chicken, 1:200, Abcam: ab41489), Brn2 (goat, 1:400 Santa Cruz: sc-6029), Ctip2 (rat, 1:500, Abcam: ab18465), FoxG1 (rabbit, 1:500, Abcam: ab18259), GABA (rabbit, 1:1000, Sigma-Aldrich: A2052), GAD67 (mouse, 1:100, Millipore: MAB5406), GFAP (chicken, 1:4000, Abcam: ab4674), MAP2 (rabbit, 1:400, Millipore: AB5622), MAP2 (chicken, 1:4000, Novus Biologicals: NB300-213), Oct4 (goat, 1:200, R&D Systems, AF1759), Pax6 (rabbit, 1:1000, BioLegend: 901301), PSD-95 (mouse, 1:50, Abcam: ab2723), Satb2 (mouse, 1:200, Abcam; ab51502), S100β (mouse, 1:500, Abcam: ab11178), Sox2 (mouse, 1:200, R&D Systems: MAB2018), synaptophysin (rabbit, 1:2000, Abcam: ab32127), Tbr1 (rabbit, 1:1500, Abcam: ab31940), Tbr2 (rabbit, 1:1000, Abcam: ab23345), vGlut1 (rabbit, 1:2000, Synaptic Systems: 135303) and vimentin (mouse, 1:500, Dako: M0725). Secondary antibodies consisted of Alexa Fluor 488 (1:400), Alexa Fluor 568 (1:400) or Alexa Fluor 647 (1:200) dyes (all Thermo Fisher Scientific). The cells were imaged using an Olympus IX51 microscope with an Olympus DP30BW camera (Olympus Corporation, Hamburg, Germany), an LSM780 laser scanning confocal microscope with a Quasar spectral GaAsP detector (all from Carl Zeiss, Jena, Germany) and a Nikon A1R + laser scanning confocal microscope with an A1-DUG GaAsP Multi Detector Unit. CellProfiler^81^ and CellProfiler Analyst^82^ software were used for quantification.

### Microelectrode array measurements

Extracellular recordings were obtained with an Axion Maestro system controlled by AxIS software (Axion Biosystems, Atlanta, GA, USA) with a 12.5 kHz sampling rate. Cells were plated on CytoView MEA 12 for recording spontaneous activity development and on CytoView MEA 48 for pharmacological experiments (both from Axion Biosystems). CytoView MEA 12 and 48 plates contained 64 or 16 electrodes per well, respectively. Recordings were performed under 37 °C temperature control, and a 5% CO_2_ atmosphere was provided during measurements exceeding 10 min. Spontaneous activity was measured twice a week for 10 min for a total of 14 weeks.

For pharmacological tests, 30 min of baseline activity was measured followed by a 30 min treatment follow-up. Pharmacological experiments were performed on rat cortical networks after 22 days on MEA and for hPSC-derived networks after 29 days on MEA. The pharmacological reagents used included kainic acid (1–5 µM, Sigma), α-amino-3-hydroxy-5-methyl-4-isoxazolepropionic acid (AMPA)/kainate receptor antagonist 6-cyano-7-nitroquinoxaline-2,3-dione (CNQX, 50 µM, Abcam), N-methyl-D-aspartate (NMDA) receptor antagonist D-(-)-2 amino-5-phosphonopentanoic acid (D-AP5, 50 µM, Sigma), γ-aminobutyric acid (GABA, 10 µM, Sigma), GABA_A_ receptor antagonist gabazine (30 µM, Sigma) and voltage-gated sodium channel blocker tetrodotoxin (TTX, 1 µM, Tocris). All pharmacological agents were added into separate MEA wells in 30 µl volumes with higher concentrations, thus resulting in the final concentrations as stated above.

### Microelectrode array data analysis

Spike detection was performed according to the stationary wavelet transform-based Teager energy operator (SWTTEO) algorithm that was presented previously^83^ and revised for biological data^43^. The algorithm was implemented in a custom-made MATLAB (MathWorks) script. During initial method testing, it was confirmed that the method reliably detects low-amplitude signals typical for hPSC-derived networks and the pharmacologically evoked fast tonic spiking that can be challenging to separate from the baseline noise, as shown in the examples in Supplementary Fig. S8. In short, data were prefiltered with an elliptic bandpass filter with a 200 Hz lower passband and a 3000 Hz upper passband frequencies. The spike detection method uses a low threshold value for the initial threshold-based spike detection to detect low amplitude spikes. Here, a threshold value of 4.5 × the estimate of the noise standard deviation was applied according to previous publication^84^. Next, extra steps were performed to remove possible false positive spikes using the SWTTEO algorithm. The number of spikes extracted by threshold detection was fed to the SWTTEO analysis, and the corresponding spike list was produced. Finally, only the spikes detected by both methods were considered true positive events. Electrodes detecting >10 spikes per minute were considered active electrodes and included in the analysis.

Burst analysis was performed utilizing the R-package meaRtools^85^. For burst detection, the logISI algorithm was integrated into the analysis code^86^ with minor modifications. When calculating burst features, only bursting electrodes were concerned. Parameters were set according to the original publication except for the minimum number of spikes in the burst, which was set to 5. A single modification to the original algorithm was added to merge short bursts in cases when the computed inter-spike interval threshold was less than 100 ms. Here, a cutoff of 100 ms was applied as the minimum time required between bursts.

Connectivity analysis was performed using the correlated spectral entropy (CorSE) method described previously^47^. Functional connectivity was calculated between all electrode pairs of the MEA during the measurement period. Briefly, CorSE quantifies the synchronization of signals by correlation of the temporal changes in their spectral contents. Magnitude of correlation gives the connectivity strength. Average CorSE values are calculated from all the MEA channels to assess the overall connectivity strength of the whole network. To observe the changes in the network formation of the most robust network participants, connectivity maps were plotted for the channel pairs, which have CorSE > 0.7.

Principal component analysis (PCA) was used as previously described^87^ to segregate MEA results from different experiments. The data were derived from two differentiations of hESC line 08/023 (named hPSC 1 and 2) and one differentiation of hiPSC line 10212.EURCCs (named hPSC 3). Additionally, MEA data were derived from three independent batches of rat embryonic neurons (named Rat 1–3). From each cell batch, the developmental time point showing the most significant activity (maximum spike rate) and two of its surrounding time points were selected for PCA analysis. The selected time points (days on MEA) were 21, 24 and 28 for hPSC 1 and 2; 70, 73 and 77 for hPSC 3; 21, 24 and 28 for Rat 1 and 2; and 24, 28 and 31 for Rat 3. A total of 7 features from spike, burst and network synchronization analysis were selected. These included the mean firing rate (MFR), burst rate, burst duration, spike frequency in burst, spikes in burst, percentage of spikes in bursts and spike time tiling coefficient (STTC, using default time bin 50 msec). All values were normalized using the standard score method. PCA was performed in MATLAB and plotted against the three major principal components.

### Statistical analysis

Due to the non-Gaussian distribution of the data, the nonparametric Mann-Whitney U test was used. A p-value < 0.05 was considered significant. All statistical tests were performed with SPSS Statistics software (version 25.0). Tampere University statistician Heini Huhtala was consulted for the statistical tests.

## Supplementary information

Supplementary information

## Data Availability

The datasets generated and/or analyzed during the current study are available from the corresponding author on reasonable request. We are planning to publish the MEA data in a subsequent publication, currently in preparation.
