# Supplementary material for: Functional characterization of human pluripotent stem cell-derived cortical networks differentiated on laminin-521 substrate: comparison to rat cortical cultures
Source: Sci Rep. 2019 Nov 20;9:17125. doi: 10.1038/s41598-019-53647-8 (PMC6868015; doi:10.1038/s41598-019-53647-8)
Supplement: Supplementary file 1 — Supplementary information [file 41598_2019_53647_MOESM1_ESM.pdf]

Supplementary information

Title

Functional characterization of human pluripotent stem cell-derived cortical networks differentiated on laminin-521 substrate: comparison to rat cortical cultures

Authors

Tanja Hyvärinen[1], Anu Hyysalo[1,2], Fikret Emre Kapucu[3,4], Laura Aarnos[1], Andrey Vinogradov[1], Stephen J. Eglen[5], Laura Ylä-Outinen[1], Susanna Narkilahti[1,*]

Author affiliations

1. Faculty of Medicine and Health Technology and BioMediTech, Tampere University, Tampere, Finland
2. Institute of Biotechnology, HiLIFE, University of Helsinki, Helsinki, Finland
3. Department of Biomedicine, Aarhus University, Aarhus, Denmark
4. Danish Research Institute of Translational Neuroscience - DANDRITE, Aarhus University, Aarhus, Denmark
5. Department of Applied Mathematics and Theoretical Physics, University of Cambridge, Cambridge, United Kingdom

*Corresponding author: Susanna Narkilahti, susanna.narkilahti@tuni.fi

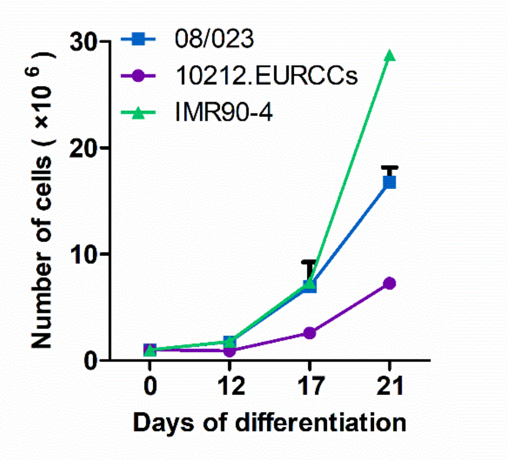

Supplementary Figure S1. **Expansion of cells on LN521 substrate during neural differentiation.** The number of cells generated with each of the hPSC lines during differentiation until cryopreservation at day 21. Results are presented relative to 1 million pluripotent cells at day 0. Data are derived from 1-3 independent differentiations and shown as the mean±s.e.m..

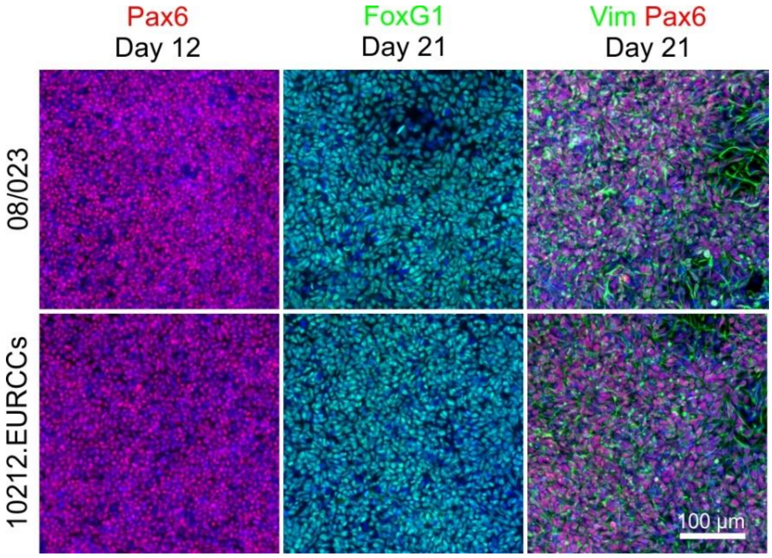

Supplementary Figure S2. **Neural differentiation on Matrigel and mouse laminin substrates.** Efficiency of neural induction on Matrigel was evaluated with immunocytochemical staining of Pax6 in cultures derived from hESC line 08/023 and hiPSC line 10212.EURCCs at day 12 of differentiation. Additionally, differentiation of NPCs on mouse laminin was assessed with FoxG1, vimentin and Pax6 staining at day 21.

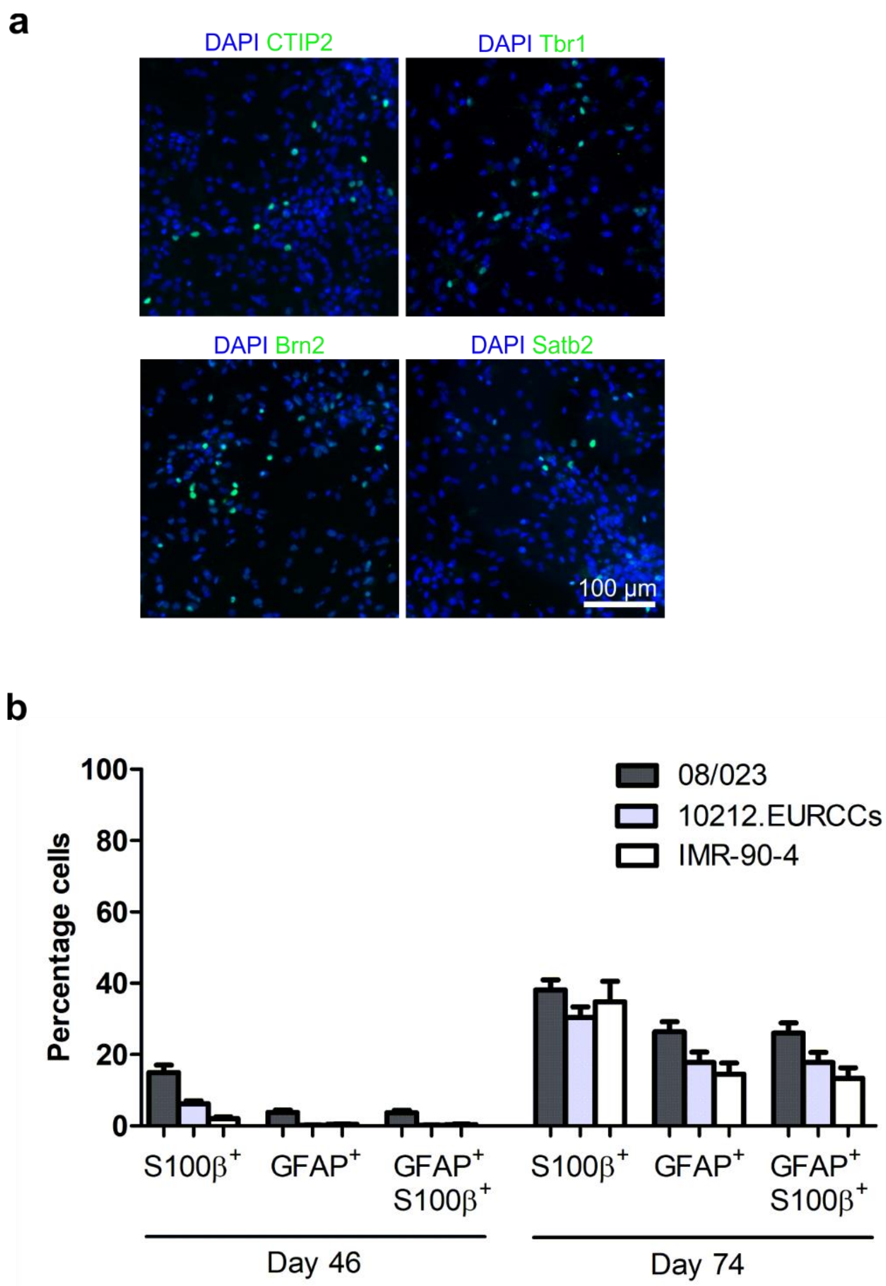

Supplementary Figure S3. **Generation cortical layer-specific neurons and astrocytes on LN521 substrate. (a)** Immunocytochemical staining verified the presence of cortical layer-specific neurons expressing the early-born deep layer markers CTIP2 and Tbr1 and the later-born upper layer markers Brn2 and Satb2 on day 74 of differentiation. Images are representative of the 08/023 hPSC line. **(b)** Neural differentiation produced astrocytes in temporal manner and most of the GFAP-positive

astrocytes were also S100β-positive in all of the studied hPSC lines. Data are presented as the mean±s.e.m. (n=9-44, data from 1-3 independent differentiations).

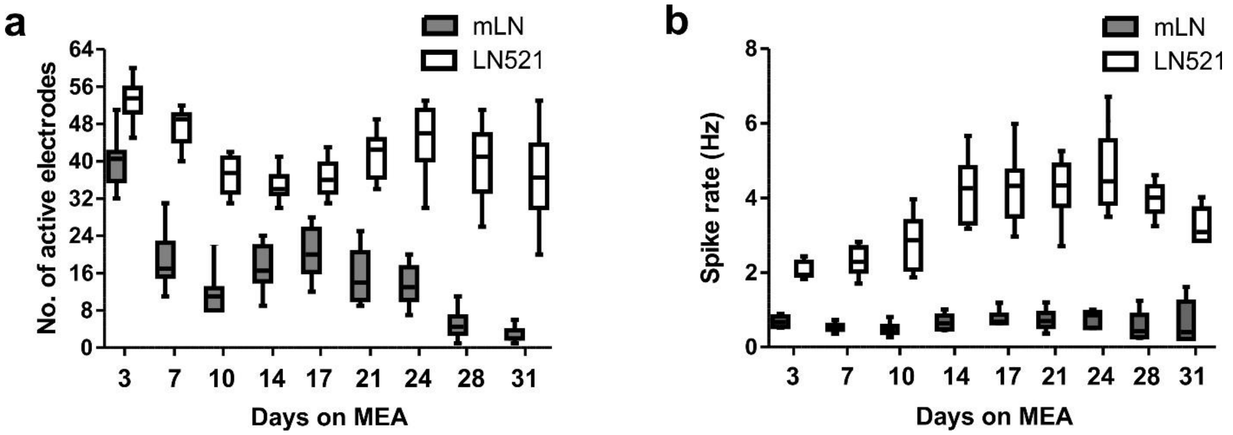

Supplementary Figure S4. **Spontaneous activity development of hPSC-derived neurons differentiated on mLN or LN521 substrates. (a)** The number of active electrodes per MEA-well (64 electrodes per well) over the measurement period. Electrodes with >10 spikes/min were considered active. **(b)** Development of **s**pike rate (Hz) per well over measurement time. Experiments were carried out with hESC line 08/023 and data consists of n=12 networks per each group. Data are presented as Tukey box plots.

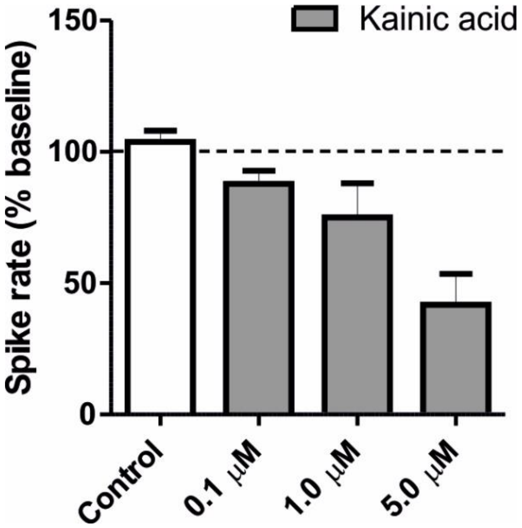

Supplementary Figure S5. **Effect of kainic acid on embryonic rat cortical networks after 22 days on MEA.** Percentage change in spike rate as compared to baseline measurement of the well. Data consists of four networks and presented as the mean±s.e.m.

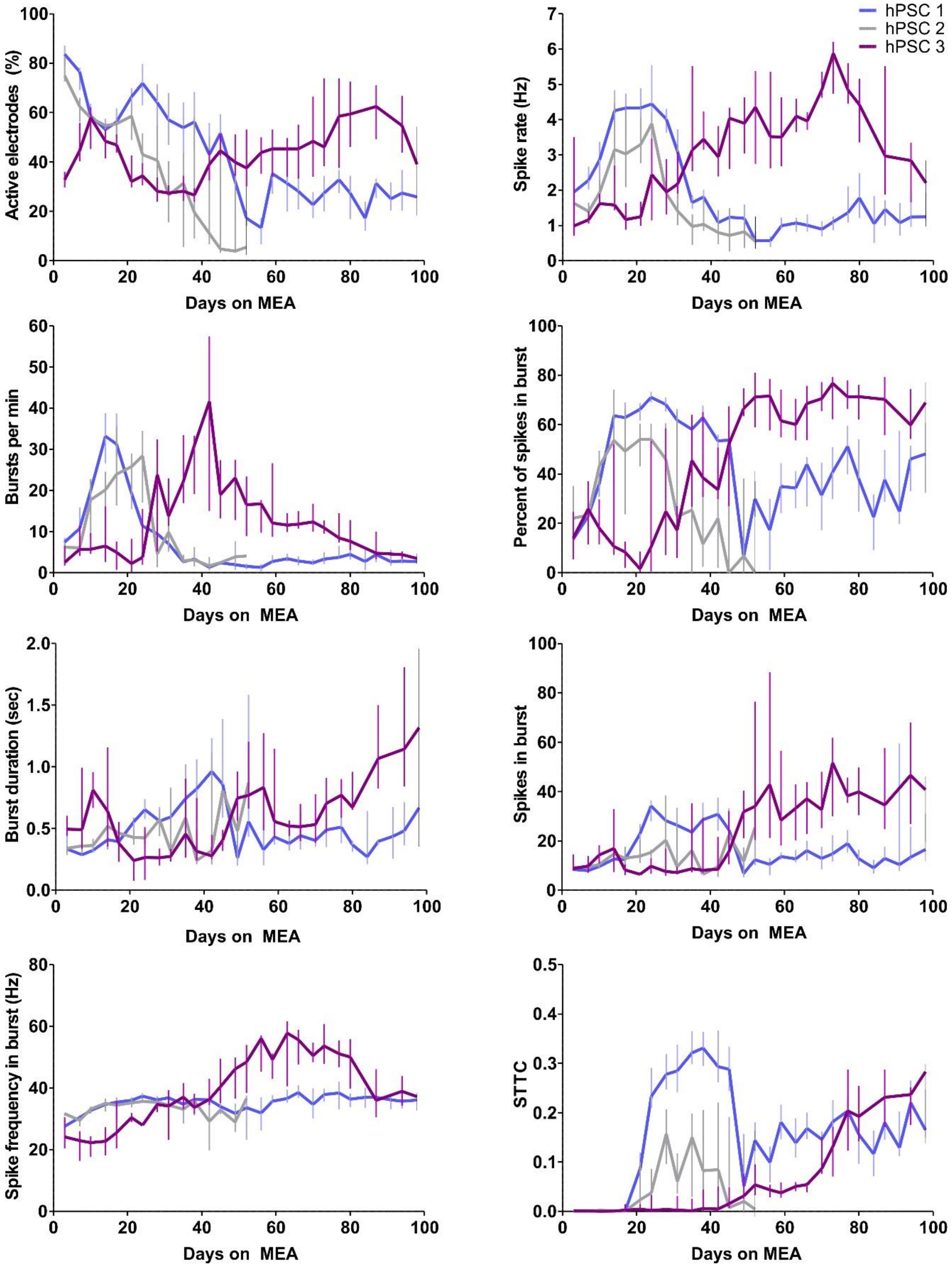

Supplementary Figure S6. **Development of spike, burst and network synchrony features over the measurement period in hPSC-derived neurons differentiated on LN521 substrate.** The number of analyzed networks are hPSC 1 (08/023) n=12, hPSC 2 (08/023) n=6, hPSC 3 (10212.EURCCs) n=4. Data are presented as the median and interquartile range.

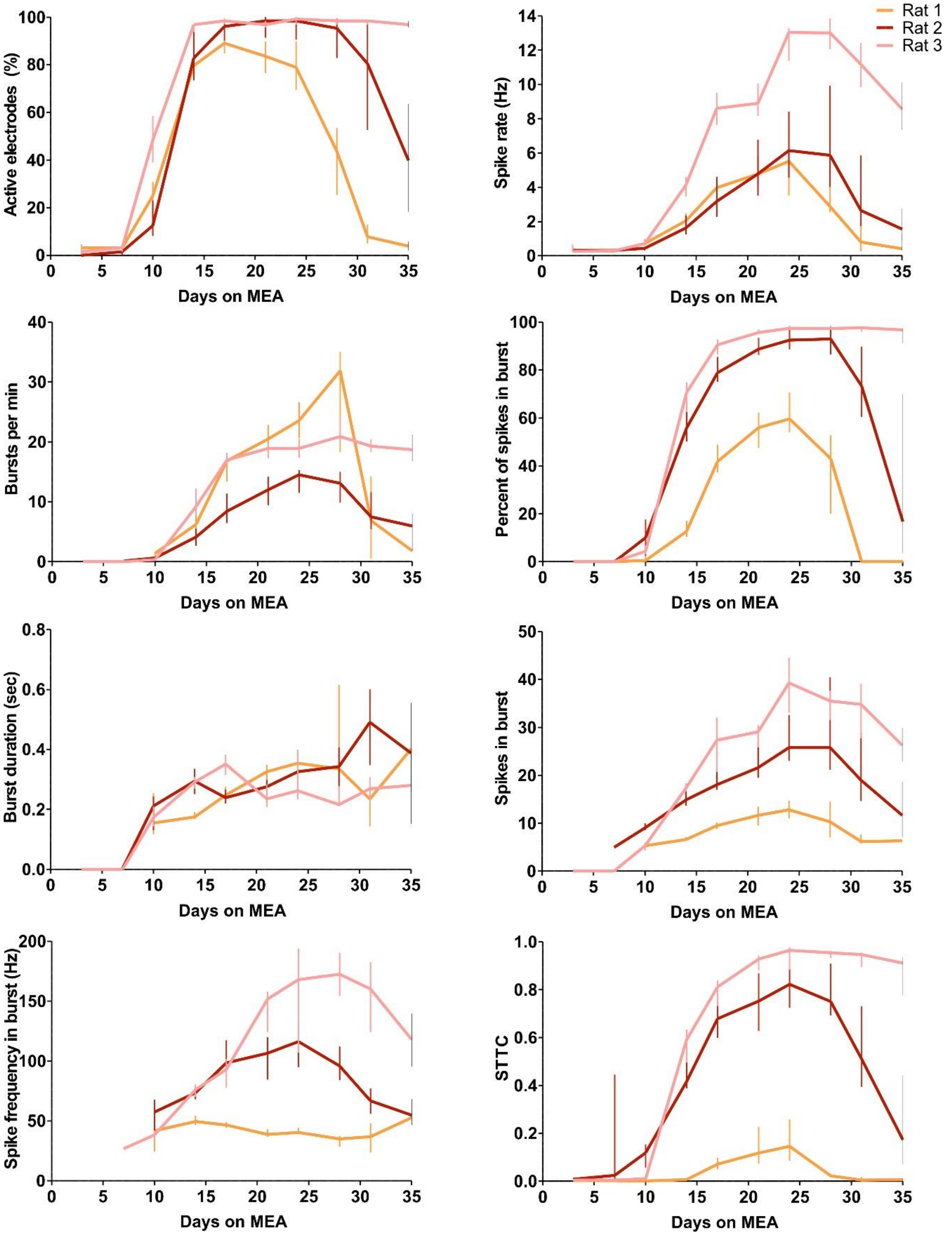

Supplementary Figure S7. **Development of spike, burst and network synchrony features over the measurement period in rat cortical neurons.** For each of the experiments (Rat 1-3) 12 networks were analyzed. Data are presented as the median and interquartile range.

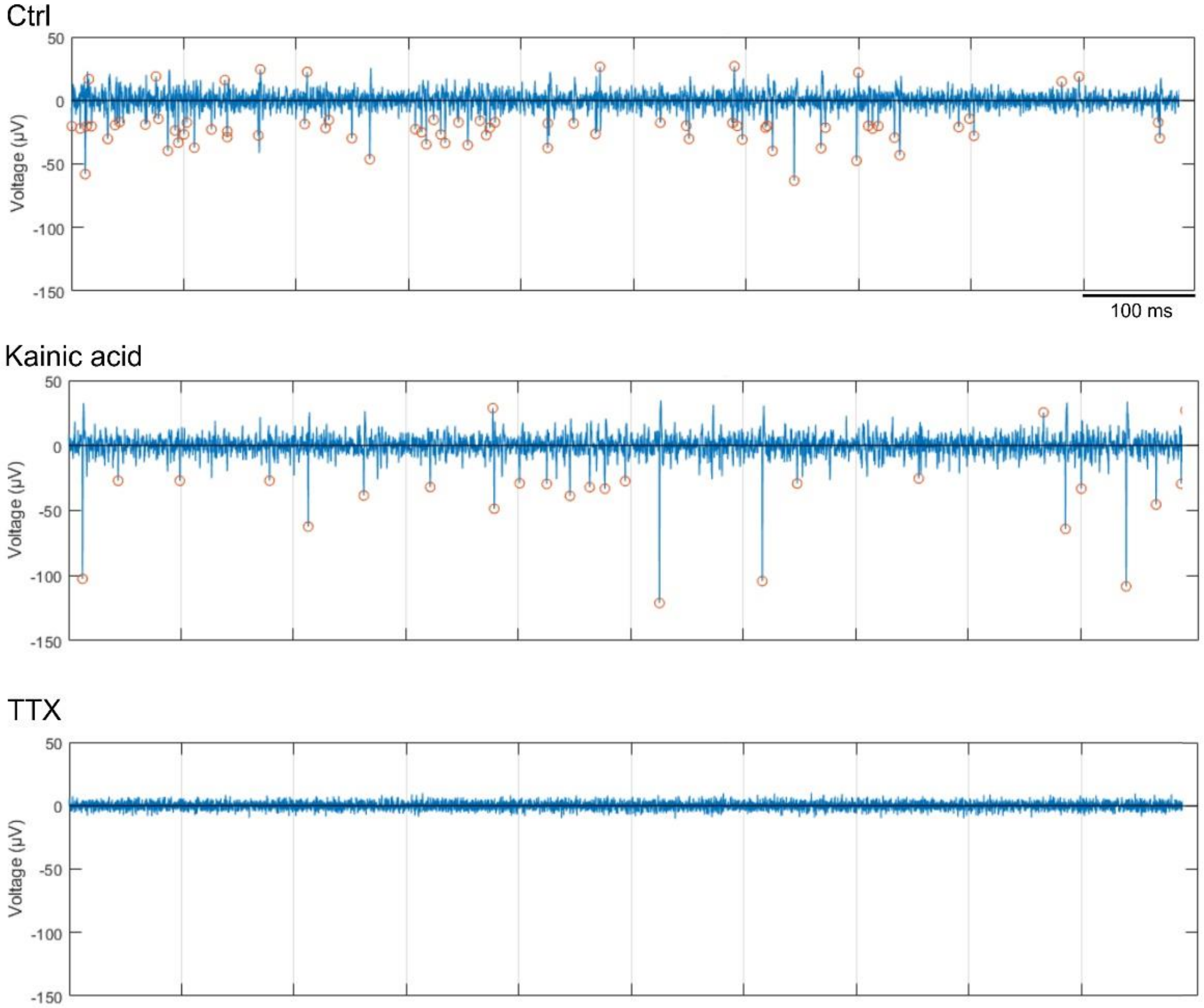

Supplementary Figure S8. **Spike detection from hPSC-derived MEA data using SWTTEO algorithm.** Detected spikes are marked with small circles in datasets showing spontaneous activity (Ctrl) and pharmacological modulation with kainic acid and TTX.
